# Expression of tumor necrosis factor-α and interleukin-1β genes in the cochlea and inferior colliculus in salicylate-induced tinnitus

**DOI:** 10.1186/1742-2094-8-30

**Published:** 2011-04-09

**Authors:** Juen-Haur Hwang, Jin-Cherng Chen, Shan-Ying Yang, Ming-Fu Wang, Yin-Ching Chan

**Affiliations:** 1 Graduate Institute of Clinical Medicine, College of Medicine, National Taiwan University, Taipei, Taiwan; 2 Department of Otolaryngology, Buddhist Dalin Tzu-Chi General Hospital, Chiayi, Taiwan; 3 School of Medicine, Tzu Chi University, Hualien, Taiwan; 4 Department of Neurosurgery, Buddhist Dalin Tzu-Chi General Hospital, Chiayi, Taiwan; 5 Department of Food and Nutrition, Providence University, Taiwan

## Abstract

**Background:**

Changes in the gene expressions for tumor necrosis factor-α (TNF-α) and/or interleukin-1β (IL-1β) during tinnitus have not been previously reported. We evaluated tinnitus and mRNA expression levels of TNF-α, IL-1β, and *N*-methyl *D*-aspartate receptor subunit 2B (NR2B) genes in cochlea and inferior colliculus (IC) of mice after intraperitoneal injections of salicylate.

**Methods:**

Forty-eight 3-month-old male SAMP8 mice were randomly and equally divided into two groups: salicylate-treated and saline-treated. All mice were trained to perform an active avoidance task for 5 days. Once conditioned, an active avoidance task was performed 2 hours after daily intraperitoneal injections of saline, either alone or containing 300 mg/kg sodium salicylate. Total numbers of times (tinnitus score) the mice climbed during the inter-trial silent period for 10 trials were recorded daily for 4 days (days 7 to 10), and then mice were euthanized for determination of mRNA expression levels of TNF-α, IL-1β, and NR2B genes in cochlea and IC at day 10.

**Results:**

Tinnitus scores increased in response to daily salicylate treatments. The mRNA expression levels of TNF-α increased significantly for the salicylate-treated group compared to the control group in both cochlea (1.89 ± 0.22 vs. 0.87 ± 0.07, *P *< 0.0001) and IC (2.12 ± 0.23 vs. 1.73 ± 0.22, *p *= 0.0040). mRNA expression levels for the IL-1β gene also increased significantly in the salicylate group compared to the control group in both cochlea (3.50 ± 1.05 vs. 2.80 ± 0.28, *p *< 0.0001) and IC (2.94 ± 0.51 *versus *1.24 ± 0.52, *p *= 0.0013). Linear regression analysis revealed a significant positive association between tinnitus scores and expression levels of TNF-α, IL-1β, and NR2B genes in cochlea and IC. In addition, expression levels of the TNF-α gene were positively correlated with those of the NR2Bgene in both cochlea and IC; whereas, the expression levels of the IL-1β gene was positively correlated with that of the NR2B gene in IC, but not in cochlea.

**Conclusion:**

We conclude that salicylate treatment resulting in tinnitus augments expression of the TNF-α and IL-1β genes in cochlea and IC of mice, and we suggest that these proinflammatory cytokines might lead to tinnitus directly or via modulating the NMDA receptor.

## Background

Tinnitus is the perception of sound in the absence of acoustic stimulation. Usually occurring together with hearing loss, tinnitus can be perceived in one or both ears, or in the head. Tinnitus is distinguishable from auditory hallucinations, which involve hearing one or more talking voices. Salicylate-induced tinnitus in mice has been a popular animal model for the study of tinnitus [1]. The mechanism of salicylate-induced tinnitus is postulated to involve accumulation of arachidonic acid caused by inhibition of cyclooxygenase (COX) that could potentiate *N*-methyl *D*-aspartate receptor (NR) currents at synapses between inner hair cells and dendrites of the cochlear spiral ganglion neuron [2].

Tinnitus-related changes in gene expression have been recently reported. Microarray studies have revealed that 87 genes are up-regulated and 140 genes are down-regulated by two-fold or more in mouse cochlea during salicylate ototoxicity [3]. Jia and Qin [4] have reported that expression of c-fos and NR2A increases in auditory cortexes of rats that experience tinnitus after salicylate injection. We also have found that mRNA expression levels for COX-2 decrease slightly, whereas expression levels of NR2B increase moderately in cochlea and midbrain of salicylate-treated, senescence accelerated prone mice substrain 8 (SAMP8 mice) [5].

Recent studies have shown that inflammatory responses occur in the inner ear under various damaging conditions, including overstimulation with noise [6] and cisplatin-induced ototoxicity [7]. However, an association between proinflammatory cytokines and tinnitus has been rarely reported. In chronic tinnitus sufferers, a relaxation training program can result in significantly decreased stress, anxious depression, anger, and tinnitus disturbance, paralleled by a reduction of TNF-α, but not IL-6 or IL-10 [8]. Also, previous studies have shown that TNF-α and IL-1β might interact with the NR [9-12] in inflammatory hyperalgesia [13], bone cancer pain [14], seizures [15], vision loss [16], and spinal cord injury [17]. Because the NR is also linked to tinnitus in rats [2] and mice [5], we hypothesized that tinnitus may be associated with changes in gene expression of these proinflammatory cytokines.

Apart from the "theory of cochlear origin" [18], tinnitus is most commonly believed to originate from the central nervous system. Positron emission tomography has shown increased metabolic activity consistent with neuronal activation in the inferior colliculus (IC) and in the auditory cortices of rats with salicylate-induced tinnitus [19]. Thus, we sought to assess gene expressions in cochlea and IC for this study.

## Methods

### Animals

Because tinnitus is most prevalent in older humans, we used the SAMP8 mice for this study, although other strains of rodents, cats, or rabbits could also have been used. Forty-eight 3-month-old male SAMP8 mice, weighing between 22 and 33 g, were randomly and equally divided into two groups: salicylate-treated (n = 24) and saline-treated (n = 24). Our Institutional Animal Care and Use Committee approved the protocols used in this study.

### Behavioral measurement of tinnitus score

All mice were trained to perform an active avoidance task, which was performed in a conditioning box with an electrical floor and a climbing pole, according to the design of Guitton et al. [2].

### Conditioning to the task

The conditioning paradigm consisted of 6 sessions performed daily for 5 days (day 1 to 5). Each session lasted 15 to 20 min, and there were 10 trials per session. Inter-trial intervals were at least 1 minute. For each trial, the conditioning stimulus was a 50 dB sound pressure level (SPL) pure tone with a frequency of 10 k Hz and a 3-second duration. The unconditioned stimulus was a 3.7 mA electric foot-shock presented for up to 30 seconds, as described by Guitton's protocol [2], by adjusting the electric voltage with fixed copper wire resistance on the floor. The time between the conditioned stimulus and the unconditioned stimulus was 1 second. The mice would climb up the pole to a safe area after the coupled conditioned and unconditioned stimuli. Electrical shocks were stopped by the experimenter when the animal climbed correctly. The "true-positive" score was the level of performance as assessed by the number of times the mice climbed correctly in response to sound. Mice were considered to be conditioned when the "true-positive" score reached at least 80% in three consecutive sessions. Only conditioned mice entered the tinnitus experiments.

### Induction and testing of tinnitus

Once conditioned, the mice rested for 1 day (day 6). Then, for 4 consecutive days (days 7 to 10), an active avoidance task was performed 2 hours after intraperitoneal injections of saline either alone or containing 300 mg/kg sodium salicylate (Sigma, St. Louis, MO) [1,2,20]. The active avoidance task consisted of one session with 10 trials. To compensate for hearing loss (threshold elevation of 15 to 20 dB SPL during 4-days injections in our preliminary, unpublished data) induced by salicylate injection, the intensity of sound that elicited the behavioral responses was increased to 70 dB SPL for the salicylate-treated group. By doing so, the sound sensation levels of all mice in both groups were similar.

During testing, a sound of 3-second duration was given and the mice were observed for another 5 seconds to see whether they would climb to the safe area as conditioned (true-positive). If the animals stayed in the safe area ≥10 seconds, the mice were returned to the floor for ongoing observation. If the mice did not climb to the safe area in response to the sound, an electrical shock was given by the experimenter to remind the mice to climb to the safe area. Again, if animals stayed in the safe area ≥10 sec, the mice were returned to the floor for ongoing observation. Finally, the experimenter observed the total number of times (tinnitus score) the mice climbed during the inter-trial silent period of 1 minute (false-positive climbs) for 10 trials.

### Samples isolation and RNA extraction from cochlea and IC

The body weights of all mice were measured at day 7 before salicylate or saline injection, and at day 10 before the mice were euthanized by decapitation. The weights of the midbrain including IC were also measured before dissection. Paired samples of cochlea and IC were immediately dissected using a Zeiss stereomicroscope and stored in a -80°C freezer until use. RNA isolation was performed using RNA-Bee isolation reagent (Friendswood, USA) with a tissue homogenizer according to the manufacturer's protocol. RNA quality was assessed using an Agilent Bioanalyzer 2100 and the ratio of absorbance measurements at 260 and 280 nm.

### Reverse transcription-polymerase chain reaction (RT-PCR)

High quality RNA was used as substrate to synthesize cDNA by reverse transcription using a MasterAmp™ High Fidelity RT-PCR Kit (Epicentre Biotechnologies, USA) in a P × 2 Thermal cycler (Thermo Electron Corporation Bioscience Technologies Division, USA). RT was carried out at 37°C for 1 hour. For PCR amplification, 7.5 μL cDNA and primers were used according to the supplier's instructions. The primers were: TNF-alpha-F, 5'-CCCCTCAGCAAACCACCAAG-3', TNF-alpha-R, 5'-CTTGGCAGATTGACCTCA GC-3'; IL-1beta-F, 5'-GAGTGTGGATCC CAAGCAAT-3', IL-1beta-R, 5'-CTCAGTGCAGGCTATGACCA-3'; NMDA receptor subtype 2B (NR2B)-F, 5'-TCC GCC GAG AGT CCT CCG T-3', NR2B-R, 5'-CTG CGT TGC CCT CGA TGT T-3'; β-actin-F, 5'-CCACACCCGCCACCAGTTCG-3', and β-actin-R, 5'-CCCATTCCCACCATCACACC-3' (Protech-taiwan, Taiwan).

The thermal cycling conditions for PCR were adjusted to the following: 3 min initial set-up at 95°C; followed by 50 cycles of denaturation (45 s at 95°C for all genes), annealing (45 s at 53°C for TNF-alpha, 52°C for IL-1beta, 54°C for NR2B, and 50°C for β-actin. A final 10 min extension at 72°C was included for all thermal cycle runs.

### Quantification of PCR products

The DNA products were measured using a Mini Horizontal Electrophoresis System (MJ-105/MP-100, Major Science, Taiwan) and an E-Box-1000/26M Inspection Certificate & Analysis Systems (E-Box Spp-010 E-capt soft ware, USA). The expression levels of TNF-α, IL-1β and NR2B genes are presented as relative ratios in comparison to β-actin.

### Statistical analysis

Data are presented as mean ± standard deviation (SD), unless indicated otherwise. Expression levels for the TNF-α or IL-1β genes, presented as ratios relative to β-actin, were compared for both groups using Student's t-test. The correlation between the tinnitus score (dependent variable) and TNF-α or IL-1β gene (independent variable) expression, and between NR2B (dependent variable) and TNF-α or IL-1β gene (independent variable) expression, were analyzed using a linear regression model. All of the above analyses were performed using the commercialized software "STATA10", and *p *values < 0.05 were considered statistically significant.

## Results

The body weight of each mouse did not change significantly during active avoidance task conditioning when performed according to Guitton's design [2]. The mean body weight before injection of intraperitoneal salicylate was 28.4 ± 1.9 g for the salicylate group and 29.0 ± 1.2 g for the control group (p = 0.1969). On day 4 of tinnitus induction, mean body weights were still not significantly different (p = 0.255) between salicylate-treated mice (29.4 ± 1.24 g) and control mice (28.8 ± 1.9 g). In addition, the weights of the midbrain including IC were not significantly different between the two groups (0.3 ± 0.03 g salicylate group, 0.3 ± 0.02 g control group, p = 0.6895).

Tinnitus scores, defined as the total number of times the mice climbed to the safe area during the inter-trial silent period of 1 minute (false positive climbs) for 10 trials, increased between day 1 and day 4 of intraperitoneal injections for salicylate-treated mice but did not increase in the control group. The mean tinnitus score at day 4 was 0.5 ± 0.5 for the control group and was 8.0 ± 1.5 for the salicylate group.

**Figure 1:**
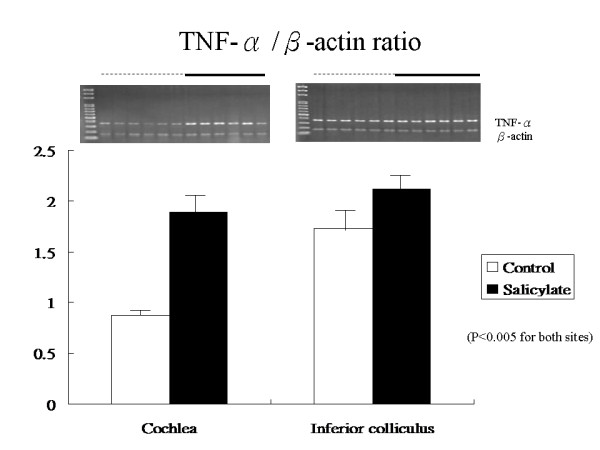
**Gene expression levels for TNF-α at day 4 after salicylate injection**. mRNA levels increased significantly in both cochlea and IC in the salicylate-treated group. Values are shown mean ± SD.

**Figure 2:**
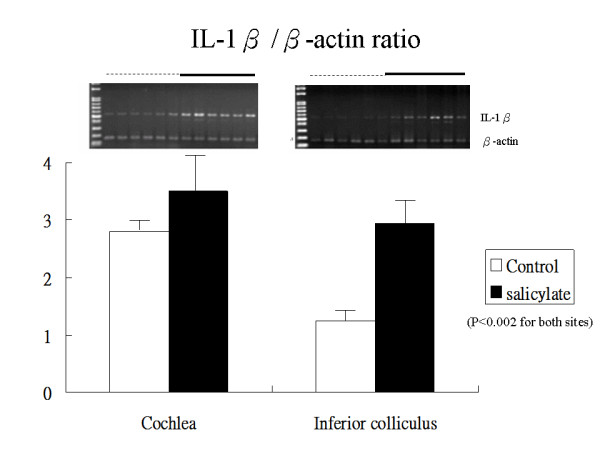
**Gene expression levels for IL-1β at day 4 after salicylate injection**. mRNA levels increased significantly in both cochlea and IC in the salicylate-treated group. Values are shown as mean ± SD.

Figure 1 shows the gene expression levels for TNF-α in cochlea and IC at day 4 after tinnitus induction. in the salicylate group compared to the control group, the mean expression level for TNF-α increased significantly in cochlea (1.89 ± 0.22 vs. 0.87 ± 0.07 respectively, p < 0.0001) and in IC (2.12 ± 0.23 vs. 1.73 ± 0.22 respectively, *p *= 0.0040). Figure 2 shows expression levels for the IL-1β gene in cochlea and IC at day 4 after tinnitus induction. In the salicylate group compared to the control group, the mean expression level for the IL-1β gene increased significantly in cochlea (3.50 ± 1.05 vs. 2.80 ± 0.28, respectively, p < 0.0001) and in IC (2.94 ± 0.51 vs. 1.24 ± 0.52, p = 0.0013).

Table 1 addresses the relationships between tinnitus scores and gene expression levels for TNF-α, IL-1β, and NR2B. Linear regression analysis showed that tinnitus scores were positively associated with gene expression level of NR2B and with gene expression levels of TNF-α and IL-1β in cochlea and IC (see Table 1 for data).

**Table 1:** Relationship between tinnitus scores and expression levels of TNF-α, IL-1β, and NR2B genes, as assessed by linear regression analysis

Genes and sites	Coefficient	Standard error	95% Confident interval	P value
***TNF-α***				
Cochlea	6.76	0.49	5.78 ~ 7.74	<0.001
IC	8.79	1.53	5.69 ~ 11.88	<0.001

***IL-1β***				
Cochlea	2.11	0.65	0.80 ~ 3.42	0.002
IC	3.14	0.38	2.38 ~ 3.91	<0.001

***NR2B***				
Cochlea	4.13	0.46	3.20 ~ 5.06	<0.001
IC	2.82	0.92	0.97 ~ 4.67	0.004

In addition, linear regression analysis showed that the gene expression level of TNF-α was positively associated with NR2B gene expression in cochlea (β ± SE = 1.07 ± 0.14, P < 0.001) and in IC (β ± SE = 0.93 ± 0.28, P = 0.001). Similarly, the gene expression level of IL-1β was positively associated with NR2B gene expression in IC (β ± SE = 0.24 ± 0.083, P = 0.005), but not in cochlea (β ± SE = 0.08 ± 0.135, P = 0.575).

## Discussion

Considering the results of Weber et al. [8], we suggested that proinflammatory cytokines, in addition to their contributions to hearing impairment [6,7], might also play an important role in the pathophysiology of tinnitus. This experimental study showed that mRNA levels of the TNF-α and IL-1β genes increased both in cochlea and IC after intraperitoneal injection of high dose salicylate. In addition, the tinnitus scores showed significant positive associations with the gene expression levels of TNF-α, IL-1β, and NR2B in both cochlea and IC.

Previous studies have focused on the roles of proinflammatory cytokines in hearing impairment. For example, virally encoded macrophage inflammatory proteins appear to play a significant role in cytomegalovirus-related hearing loss [21]. Cochlear immunohistochemistry has confirmed the presence of numerous cytokines, as well as NF-kB, in otitis media-induced cochlear cytotoxicity [22]. TNF-α, IL-1βa and IL-6 are significantly induced in the lateral side of the spiral ligament and stria vascularis early after noise exposure. Furthermore, IL-6 is detected in spiral ganglion neurons at 12 and 24 hr after noise exposure. These cytokines may initiate an inflammatory response and lead to noise-induced cochlear damage [6,23]. In addition, TNF-α, IL-1β and IL-6 are associated with cisplatin-induced ototoxicity [7]. However, infusion of TNF-α into the cochlea of guinea pigs does not result in obvious hearing loss, although inflammatory cells are recruited to the cochlea [24]. The results of this study, together with those of Weber et al. [8], suggest that proinflammatory cytokines might also account for the induction of tinnitus, but the exact mechanisms involved are still unclear.

Tinnitus can be regarded as phantom auditory pain, which is often followed by peripheral hearing loss [25]. In chronic neuropathic pain studies, TNF-α, IL-1β and IL-6 have been reported to induce central sensitization and hyperalgesia after nerve injury via distinct and overlapping synaptic mechanisms in superficial dorsal horn neurons. Furthermore, these proinflammatory cytokines may induce long-term synaptic plasticity through CREB-mediated gene transcription [26-30]. It is reasonable to hypothesize that these cytokines might also contribute to tinnitus via mechanisms similar to those of post-traumatic neuropathic pain.

Tinnitus has been reported to be highly associated with NMDA receptor activity [2] and/or its gene expression [5]. Several studies have reported an interaction between TNF-α and/or IL-1β and functions of NMDA receptor [9-17]. Conversely, NMDA can also increase expression of TNF-α and IL-1β, as well as endothelial adhesion molecules, including ICAM-1, and induce leukocyte accumulation in retinal vessels and retina ganglion cell death [16]. Glutamate could influence the release of cellular proinflammatory responses in arthritis [12]. Therefore, it is also reasonable to hypothesize that TNF-α and IL-1β might contribute to tinnitus via augmenting NMDA receptor expression and/or its functions.

## Conclusion

These data demonstrate that salicylate-induced tinnitus correlates with increased gene expression of TNF-α and IL-1β. The tinnitus scores of salicylate-treated mice showed significant positive associations with the expression levels of the TNF-α and IL-1β, and NR2B genes. Furthermore, the gene expression levels of TNF-α and IL-1β correlated positively with that of the NR2B gene. We suggest that these proinflammatory cytokines might lead to tinnitus directly or via modulation of NR2B gene expression in salicylate-induced tinnitus.

## Competing interests

The authors declare that they have no competing interests.

## Authors' contributions

JHH conceived and designed the study. SYY carried out the laboratory experiments. JHH and YCC performed the statistical analysis. JHH drafted the manuscript. JCC, MFW and YCC supervised the study. All authors participated in the critical revision of the manuscript. All authors have given final approval of the manuscript to be published.
